# The effect of joint orientation on passive movement of the dog’s stifle

**DOI:** 10.3389/fvets.2023.1207164

**Published:** 2023-06-28

**Authors:** Nadav Yair, Christos Yiapanis, Ron Ben-Amotz, Joshua Milgram

**Affiliations:** ^1^The Robert H. Smith Faculty of Agriculture, Food and Environment, The Laboratory of Biomechanics, Koret School of Veterinary Medicine, Hebrew University of Jerusalem, Jerusalem, Israel; ^2^Cyvets Veterinary Centre, Paphos, Cyprus

**Keywords:** stifle, passive motion, biomechanics, *in-vitro*, canine, dog

## Abstract

**Introduction:**

The cranial cruciate ligament (CCL) is one of numerous structures which determine the path of the tibia relative to the femur when passively flexing/extending the stifle of the dog. The effect of cutting the CCL on passive motion with the hind limb in different orientations, is unknown. The aim of this study was to describe passive movement of the tibia relative to the femur in dogs, with the hind limb in three different orientations, and with CCL intact and cut.

**Methods:**

Ten cadaveric hind limbs were obtained from dogs weighing between 20 kg and 25 kg and prepared for testing in a custom-built joint testing machine. Each hind limb was tested in three different orientations with data collected, using an electromagnetic tracking system, during 2 cycles of flexion/extension with the CCL intact and cut. Each cycle was initiated with the stifle in full extension (0°) and data was collected at 0°, 20°, 30°, 40°, 45°, and 55° of stifle flexion/extension.

**Results:**

Flexion of the stifle resulted in caudal translation and internal rotation of the tibia relative to the femur, with cranial translation and external rotation occurring during extension along the identical path. Cutting the cranial cruciate ligament did not result in significant differences in translation or rotation when the stifle was orientated to approximated the standing position of a dog.

**Discussion:**

Isometric points at the origin and insertion of the CCL can potentially be identified in CCL deficient stifles using a technique based on passive motion of an intact stifle.

## 1. Introduction

The cranial cruciate ligament (CCL) is clinically the most important structure in the canine stifle due to the high incidence of rupture, and the resulting joint instability and loss of function. (1–5). It has been shown that in spite of clinical improvement, current techniques for the repair of the CCL do not completely stabilize the joint with persistent residual instability (6, 7). Progression of degenerative joint disease after surgical management has been attributed to several factors, however, residual instability, which is likely a major contributing factor to the progression of degenerative joint disease, has not been assessed (8–11). Ideally, the surgical procedure to repair a ruptured cranial cruciate ligament should restore normal biomechanics of the stifle, as well as provide an excellent clinical outcome.

Normal movement and load transmission through the stifle of dogs is dependent on joint morphology, articular cartilage and supporting bone, menisci, ligaments, and muscle/tendon units (1, 12). It has been shown in 3D kinematic studies (13–16) and *in-vitro* biomechanical studies (17–22) that the primary rotations of the dogs stifle occur in two planes with the tibia rotating internally relative to the femur as the stifle is flexed. The coupling of internal/external rotation of the tibia to flexion/extension of the knee joint is referred to as the “screw-home” movement of the stifle. Despite advances in the understanding of the biomechanics of the dogs stifle, theoretical models which form the basis of the TPLO and TTA procedures are based on the human knee (23, 24). In people the basic kinematic principles of the knee joint have been described using loaded and passive motion studies to describe the movement of the tibia relative to the femur during flexion and extension of the knee joint (25–30). It was shown experimentally, that when passively flexing normal cadaveric knees of people the tibia rotates internally as the knee is flexed. In addition, the movement path of the knee in flexion is virtually the same as the movement path in extension, and the tibia rotates externally as the tibia is extended (27).It was also shown, in the same study, that when applying a force to the joint to cause a deviation to the normal path, the joint snaps back to the original path when the force is removed (27).

The aims of surgical intervention to repair a ruptured cranial cruciate ligament in dogs are to restore normal stifle biomechanics, and to achieve an acceptable clinical outcome. Intuitively, replacing a torn CCL will transmit the same load and perform the same guiding and restraining functions as it did in the intact joint. There are many challenges to overcome before intraarticular repair can be considered a viable method for the repair of the CCL deficient stifle of the dog (31–34). One of the challenges is the identification of isometric points for placement of the graft/prosthesis, a task made more challenging by the large range in stifle conformation and biomechanics between breeds of dogs (16). We have recently described a method, using 3D modeling and passive motion of the stifle, to identify the location of the isometric components of the CCL at the origin on the femur and insertion on the tibia (35). Due to the numerous structures which determine the path of the tibia relative to the femur, we hypothesise that rupture of the CCL is unlikely to affect the passive motion of the tibia relative to the femur. This would allow surgical planning on the effected leg and would be useful in cases of bilateral CCL rupture. The objective of this present study was to describe the passive motion of the stifle joint, with the stifle in three different orientations, and with the CCL intact and cut.

## 2. Materials and methods

### 2.1. Samples

Ten hind limbs were harvested from 10 skeletally mature dogs with a mean weight of 30 kg (Range 29 kg – 31 kg), and the mean tibial length (lateral malleus to lateral tibial condyle) was 17.6 cm (range 17.0 cm–18.5 cm). The specimens were obtained from dogs under 2 years of age, free of locomotor deficits and euthanized for reasons unrelated to this study. All dogs were donated to this study with signed owner consent and permission to perform this study was granted by the institutional animal care and use committee (#KSVM VTH/31_2019). Specimens were only included in this study after the stifle was confirmed to be free of pathology on two orthogonal radiographic views. Once the status of the stifle was confirmed the hind limb was harvested by dislocation of the coxofemoral joint, wrapped in saline soaked towels, and stored at −20° C.

### 2.2. Description of experimental equipment

**Figure 1:**
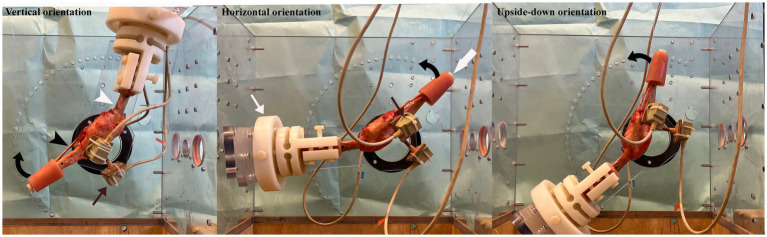
Figure showing the hind limb in the joint testing machine (partially dismantled so as not to obstruct the view of the specimen) with the femur (white arrow head) orientated vertically, horizontally and upside-down. The femur was potted in polymethylmethacrylate (PMMA) and secured in a clamp (thin white arrow) which was bolted into the joint testing machine. The tibia (black arrow head) was free to move and the stifle was flexed (direction of curved black arrow) and extended by pulling on a strand of 100 lbs. fishing line, passed over a pulley above the joint testing machine and attached to bolt (thick white arrow) secured in the PMMA of the distal tibia. Three sensors (thin black arrows) were attached to the tibia to determine the movement of the tibia relative to the femur as the stifle was flexed.

The custom-built joint testing machine and clamp was manufactured entirely from non-ferromagnetic materials, and in this study the proximal femur was rigidly secured in the clamp. The orientation of the hind limb in the joint testing machine could be altered, and each specimen was tested with the femur at -15° (Verticle), 90° (Horizontal), and 135° (Upside-down). The orientations of the hind limb in the joint testing machine were selected such that the stifle was in full extension under gravity (Figure 1). The testing machine permitted the joint flexion/extension angle to be set in increments of 5° by moving the bone distal to the joint. In this study the tibia was moved to flex/extend the stifle and measurements were taken at 0°, 20°, 30°, 40°, 45°, and 55° of stifle flexion/extension.

The orientation of the hind limb in the joint testing machine was standardized prior to testing using a custom manufactured alignment chamber. During alignment the orientation of the femur in the clamp was adjusted until bone landmarks on the femur were aligned, in orthogonal radiographic views, with markers embedded in walls of the alignment chamber. Once the desired position of the femur was achieved, the clamp was locked which prevented further movement of the femur relative to the clamp. The movement of the tibia relative to the femur was determined using an electromagnetic tracking system (‘Flock of Birds’ electromagnetic tracking system, Ascension Technology Inc., Burlington, Vermont, United States).

### 2.3. Sample preparation

The hind limb to be tested was thawed overnight at room temperature and once fully thawed the specimen was stripped of soft tissue leaving the periarticular soft tissues of the stifle intact. The femur was cut in the transverse plane at the mid diaphysis, the tibia was cut in the transverse plane 13 cm from the level of the joint and both cut ends were potted in polymethylmethacrylate (PMMA) using a custom-made mould. Three bone tunnels (diameter 4.0 mm) were drilled at the cranial, medial and lateral aspects of the tibial plateau for the connection of the sensors. The tunnels were initiated at the insertion of the patella tendon on the tibial tuberosity, and at the medial and lateral aspects of the medial and lateral tibial condyles, respectively. The potted end of the femur was secured in the clamp, bolted into the alignment chamber, and aligned as described above. Once aligned, the specimen and clamp were removed from the alignment chamber and sensors attached to wooden dowels were inserted into the bone tunnels. Care was taken to ensure that there was no movement between the sensors and the tibia.

### 2.4. Points of interest and definition of axes

**Figure 2:**
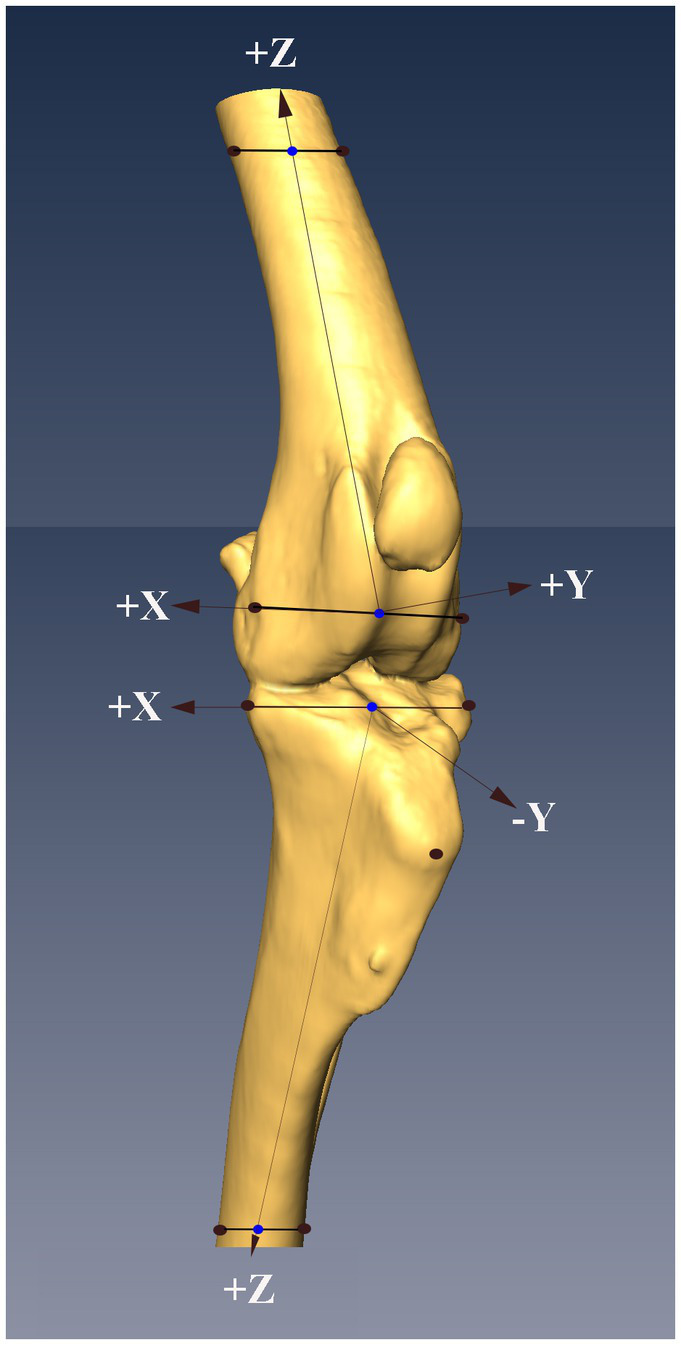
Figure showing the locations of the nine points of interest (POIs, black dots) on the femur and tibia, and the system of axes defined based on the POIs (see text for details). The midpoints of the lines between the proximal and distal points on the femur and tibia were determined (blue dots), with the midpoints on the distal femur and proximal tibia serving as the origins of the system of axes embedded in each bone. The z-axis in both bones were defined as the line that passed through the origin of the system of axes and the midpoint (blue dots) between the POIs at the other end of the bone.

Prior to testing at each of the orientations, the location of the hind limb within the magnetic field generated by the Nest of Birds was defined by collecting the coordinates of 9 points of interest (POIs) (Figure 2). The coordinates of these points were collected from each hind limb with the stifle in full extension under gravity. Four POIs were selected on the femur and 5 POIs were selected on the tibia (Figure 2) with the POIs coinciding with prominent bone landmarks where possible. In the absence of bone landmarks, POIs were marked on the cortex of the bone with a permanent marker to ensure they could be easily identified.

The POIs on the femur were the lateral and medial epicondyles distally and two points marked on the medial and lateral aspect of the proximal femoral cortex distal to the PMMA. The points selected on the proximal tibia were the lateral aspect of the lateral tibial condyle, the medial aspect of medial tibial condyle and the center of the insertion of the patella tendon on the tibial tuberosity. The points selected on the distal tibia were two points marked on the lateral and medial aspects of the distal tibial cortex proximal to the PMMA. The coordinates of the POI on the femur and tibia were collected using a sensor rigidly attached to a stylus. The stylus was calibrated prior to the testing of each specimen. The point of the stylus was placed on each POI and while in contact with the POI a stream of positional data (>200 lines of data) was collected from the sensor.

Four points of interest on the femur were used to define a system of axes embedded in the femur (Figure 2). The origin of the system of axes was defined as the center of the line connecting the 2 distal points. The *z*- axis was defined as a line connecting the origin of the system of axes and the center of the line between the proximal femoral points. A plane was then defined using the 2 center points, previously defined, and the point on the medial femoral epicondyle. The *y*- axis was defined as a line perpendicular to the defined plane and passing through the origin. The *x*- axis was defined as a line perpendicular to the 2 previously defined axes and passing through the origin.

Four POIs on the tibia were used to define a system of axes embedded in the tibia (Figure 2). The origin of the system of axes was defined as the center of a line between the points on the lateral aspect of the lateral tibial condyle, and medial aspect of medial tibial condyle. The *z*- axis was defined as a line connecting the origin of the system of axes and the center of the line between the distal tibial points. A plane was then defined using the 2 center points, previously defined, and the point on the medial tibial condyle. The *y*- axis was defined as a line perpendicular to the defined plane and passing through the origin. The *x*- axis was defined as a line perpendicular to the 2 previously defined axes and passing through the origin.

### 2.5. Specimen testing

Each specimen was tested with the femur orientated vertical, horizontal and upside-down (Figure 1). The angle of the joint extended under gravity was measured with a hand goniometer and this angle was defined as 0°. A stream of data (>500 data points) was recorded from each of the three sensors attached to the proximal tibia with the stifle in full extension. This ensured that the coordinates of the POIs on the proximal tibia and the output of the sensors at each of the POI were collected with the tibia in the identical position. The tibia was then flexed/extended using 100 lbs. nylon fishing line passed through a tunnel in the head of a plastic bolt which was placed into a hole in the distal PMMA and over a pulley above the specimen. The cranial aspect of the PMMA (with the femur orientated vertically and horizontally) and the caudal aspect of the PMMA (with the femur orientated upside down) on the distal tibia was rested on a wooden dowel during the collection of data at each of the angles tested. Streams of data (>500 data points) were recorded from each of the three sensors attached to the tibia at stifle flexion/extension angles of 20°, 30°, 40°, 45°, and 55°. Flexion and extension data with the CCL intact were collected for 2 cycles. Once testing of the intact joint was completed, the stifle was approached via a mini medial arthrotomy and the CCL cut. The mini arthrotomy was closed with 3/0 nylon in a continuous pattern and each specimen was retested using the identical technique (2 cycles of flexion/extension at each orientation). The specimen was periodically sprayed with saline which ensured that it was kept moist throughout the period of preparation and testing.

### 2.6. Data processing

The output of the Nest of Birds consisted of the *x*, *y*, and *z* coordinates of the sensor in the coordinate system generated by the transmitter (global coordinate system), as well as a 3 × 3 rotational matrix resulting in a row of data with 12 values. Systems of axes embedded in the femur and tibia were defined using the coordinates of the POIs. Once the systems of axes were defined, the location of their origins were determined in the global coordinate system. Similarly, the locations of the sensors attached to the tibia at 0° were determined in the global coordinate system. A vector and a rotational difference matrix between the origin of the global coordinate system and the origins of the systems of axes embedded in the femur and tibia were then defined. Once these were determined, these systems of axes were aligned with the global system of axes, and using the same method location of the sensors were aligned with the relevant POIs. This enabled us to describe the translations and rotations of the system of axes embedded in the tibia relative to the system of axes embedded in the femur using anatomically meaningful directional terminology. All data manipulation was performed using commercial software with custom written code (MATLAB 7.0, The Mathworks, INC, Natick, MA United States).

### 2.7. Statistical analysis

Repeated measures ANOVA was used to assess the changes in translation and rotation which occurred during flexion/extension of the stifle with the femur at all orientations and with the CCL intact and cut. No difference was found between the 2 cycles at all orientations, with the CCL intact and cut, and all the data from each specimen was combined for the analysis. Statistical analyses were performed using statistical software (SPSS 15.0 for Windows, Chicago, IL). *p* < 0.05 was considered statistically significant.

## 3. Results

**Figure 3:**
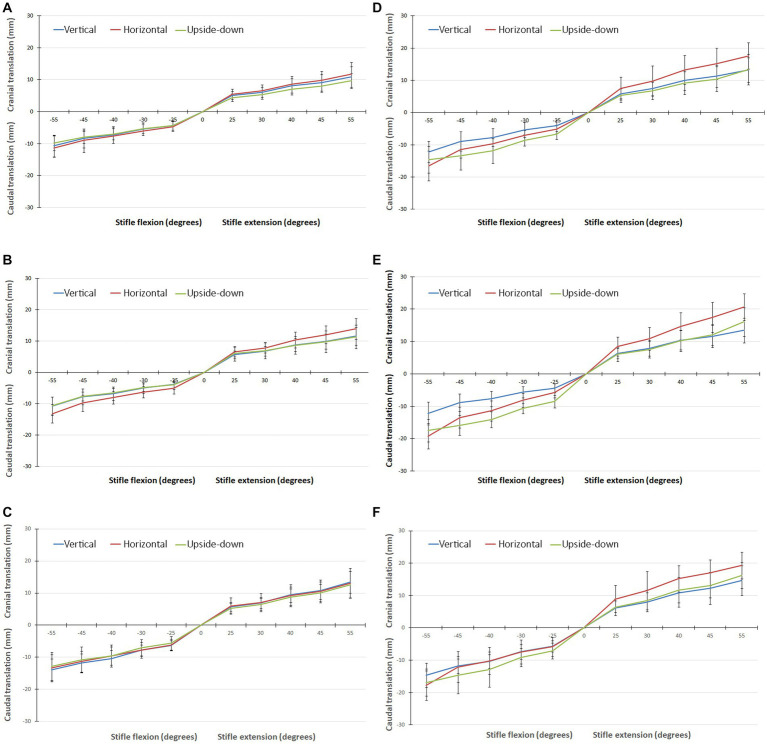
**(A)** Graph showing the translation of the origin of the system of axes, in all three orientations, with the CCL intact when flexing/extending the stifle though 55°. **(B)** Graph showing the translation of the medial tibial condyle, in all three orientations, with the CCL intact when flexing/extending the stifle though 55°. **(C)** Graph showing the translation of the lateral tibial condyle, in all three orientations, with the CCL intact when flexing/extending the stifle though 55°. **(D)** Graph showing the translation of the origin of the system of axes, in all three orientations, with the CCL cut when flexing/extending the stifle though 55°. **(E)** Graph showing the translation of the medial tibial condyle, in all three orientations, with the CCL cut when flexing/extending the stifle though 55°. **(F)** Graph showing the translation of the lateral tibial condyle, in all three orientations, with the CCL cut when flexing/extending the stifle though 55°.

The translations of the origin of the axes embedded in the tibia, and the lateral and medial condyles when flexing the stifle 55°, with the CCL intact and cut, are shown in Figure 3 and listed in Table 1. There were significant differences in the cranial/caudal translation of the origin of the system of axes and lateral and medial tibial condyles of the tibia relative to the femur as the stifle was flexed and extended (*p* < 0.0001). Changing the orientation of the stifle (*p* = 0.0012) and cutting the CCL (*p* = 0.0006) resulted in significant changes in translation of the origin of the system of axes and lateral and medial tibial condyles of the tibia relative to the femur. There was no difference in the translation during flexion and extension or between the repetitions for any of the testing conditions. With the femur orientated vertically, there was no difference in the translation of the origin of the system of axes, medial condyle and lateral condyle, with the CCL intact and cut, in both flexion and extension. With the femur orientated horizontally and upside-down there was a significant effect (*p* = 0.048) on the translation of the origin of the system of axes and lateral and medial tibial condyles of the tibia relative to the femur. In addition, with the femur orientated horizontally and upside-down, there was a significant (*p* = 0.0006) effect of cutting the CCL on the translation of the origin of the system of axes and lateral and medial tibial condyles of the tibia relative to the femur.

**Table 1:** Translation of the tibia relative to the femur when flexing the stifle 55°.

			Orientation	Movement	CCL Intact		CCL cut		Translation	*p*-value
			Mean translation (mm)	SD (mm)	Mean translation (mm)	SD (mm)				
Origin of axis	Vertical	Flexion	10.67	± 3.41	12.21	± 3.30	Caudal	NS
		Extension	10.92	± 3.19	13.21	± 3.87	Cranial	NS
	Horizontal	Flexion	11.41	± 3.88	16.51	± 4.61	Caudal	<0.001
		Extension	11.71	± 3.77	17.53	± 4.09	Cranial	<0.001
	Upside-down	Flexion	9.81	± 2.36	14.57	± 4.17	Caudal	<0.01
		Extension	9.64	± 2.28	13.33	± 4.75	Cranial	<0.01
Lateral tibial condyle	Vertical	Flexion	10.73	± 2.92	12.21	± 3.48	Caudal	NS
		Extension	11.85	± 2.82	13.43	±3.81	Cranial	NS
	Horizontal	Flexion	13.12	± 2.89	19.18	± 3.93	Caudal	<0.001
		Extension	13.95	±3.32	20.69	± 4.11	Cranial	<0.001
	Upside-down	Flexion	10.63	± 2.81	17.49	± 3.50	Caudal	<0.01
		Extension	11.33	± 3.77	16.06	± 4.38	Cranial	<0.01
Medial tibial condyle	Vertical	Flexion	13.88	± 3.43	14.64	± 3.79	Caudal	NS
		Extension	13.38	± 3.39	14.66	± 4.59	Cranial	NS
	Horizontal	Flexion	13.36	± 4.25	17.79	± 4.56	Caudal	<0.001
		Extension	13.12	± 4.65	19.26	± 4.11	Cranial	<0.001
	Upside-down	Flexion	12.87	± 4.41	16.91	± 4.19	Caudal	<0.01
		Extension	12.62	± 4.11	16.19	± 4.01	Cranial	<0.01

*p*-values are for the comparisons of translations with the CCL intact and cut (SD, standard deviation; NS, not significant).

**Figure 4:**
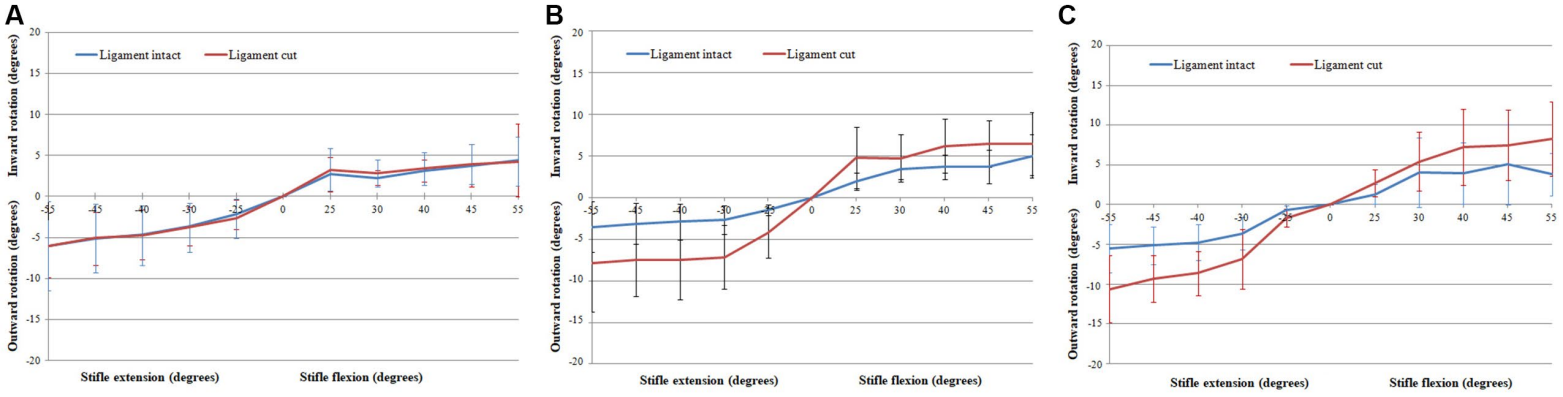
Graphs showing the Rotation of the tibia relative to the femur when flexing the stifle though 55° with the CCL intact and cut with the femur orientated vertically **(A)**, horizontally **(B)**, and upside-down **(C)**.

The rotation of the tibia relative to the femur when flexing the stifle 55°, with the CCL intact and cut, are shown in Figure 4 and are listed in Table 2. Internal/external rotation of the tibia relative to the femur changed significantly as the stifle was flexed and extended (*p* < 0.0001). However, no differences in the rotation were found when changing the orientation of the femur (*p* = 0.64), flexion/extension (*p* = 0.889), and with the CCL intact and cut (*p* = 0.5). The tibia rotated internally relative to the femur during flexion of the stifle, and externally relative to the femur during extension of the stifle.

**Table 2:** Rotation of the tibia relative to the femur when flexing the stifle 55°.

	Orientation	Movement	CCL intact		CCL cut		Rotation	*p*-value	Mean rotation (º)	SD (º)	Mean rotation (º)	SD (º)
Tibia relative to the Femur	Vertical	Flexion	6.09	± 3.74	6.04	± 5.40	Internal	NS
		Extension	4.45	± 4.46	4.24	± 2.99	External	NS
	Horizontal	Flexion	3.53	± 3.02	7.84	± 5.92	Internal	NS
		Extension	4.99	± 2.61	6.43	± 3.77	External	NS
	Upside-down	Flexion	5.53	± 3.07	10.65	± 4.21	Internal	NS
		Extension	3.82	± 2.69	8.29	± 4.65	External	NS

*p*-values are for the comparisons of rotations with the CCL intact and cut (SD, standard deviation; NS, not significant).

## 4. Discussion

In this study we characterised the translation and rotation of the tibia relative to the femur during passive flexion/extension of the stifle. We found that, with all repetitions and under all testing conditions, the tibia rotated internally relative to the femur when the stifle was flexed, and followed the same path (rotated externally) when the stifle was extended. These findings are similar to the results of passive *in-vitro* studies in dogs (18–22) and people (25–27). Stifle motion is characterised by coupling of flexion/extension and internal/external has been called the “screw-home mechanism” of the knee (27, 36). The vertical orientation of the femur was selected to approximate the standing position of the dog. Under these testing conditions the differences between the translations and rotations with the CCL intact and cut were not significant. Changing the orientation of the femur and cutting the CCL resulted in a significant increase in the translation of the tibia relative to the femur but did not cause a significant change in internal/external rotation.

With the CCL intact and with the femur orientated vertically, the tibia rotated internally 6.09° ± 3.74° when flexing the stifle 55°, and rotated externally 4.45° ± 4.46° when extending the stifle 55° (Table 2). Differences in experimental setup make it impossible to compare results between studies. An *in-vitro* passive motion study showed both internal and external rotation of the tibia relative to the femur when flexing the dog’s stifle though 120°, with a peak internal rotation of 20.0 ± 13.8° (21). Our study is based on, and our findings are similar to a passive motion study done using human cadaveric knees. In this study an internal rotation of the tibia relative to the femur of 14°– 36° was seen when flexing the knee 100° (27). It has been shown in dogs that cutting the CCL, in addition to one of the collateral ligaments, had a minor effect on rotation of the tibia relative to the femur with the stifle extended, and a more pronounced effect when the stifle was flexed (36). As expected cutting the CCL did result in an increase in internal/external rotation of the tibia relative to the femur with the femur orientated horizontally and upside-down, however, the change was not significant. Similarly, in a previously published *in-vitro* passive motion study, the effect of cutting the CCL on internal/external rotation of the tibia relative to the femur, when tested from full extension to maximum allowable flexion, was not significant (19).

We conclude that with the femur orientated vertically, cutting the CCL has the smallest effect on internal/external rotational instability of the stifle during passive motion. In this orientation the conformation of the joint, menisci and periarticular soft tissues likely play an important role in determining the path of the tibia relative to the femur. It is important to note that these results can only be obtained by passive movement, and any additional loading of the joint would likely affect the translation and rotation of the stifle. The lack of an effect on the path of the tibia relative to the femur with the CCL intact and cut, with the femur orientated vertically, can be considered a limitation of testing the stifle using passive motion. However, we have developed a method for identifying isometric points at the origin and insertion of the CCL, and on the lateral aspect of the femur and tibia using passive motion and a 3D model of the intact stifle of a dog (35). The CCL is composed of two components with only the craniomedial component remaining under tension throughout the range of motion of the stifle (37). We were able to confirm that on the femur isometric points are restricted to a part of the origin of the CCL, while on the tibia the area of insertion is large. Findings of this study suggest that passive motion can be used to identify isometric points in the affected limbs of clinical cases of CCL rupture. We speculate that the individualized identification of isometric points, will improve the clinical outcome of dogs treated with intra-articular and extra-articular repairs of the CCL.

With the femur orientated vertically, differences in the translations of the origin of the system of axes, medial condyle and lateral condyle, with the CCL intact and cut were not significant. Caudal translation of the origin of the system of axes, lateral tibial condyle and medial tibial condyle during 55°of flexion were 10.67 mm ± 3.41 mm, 10.73 mm ± 2.92 mm, and 13.88 mm ± 3.43 mm, respectively (Table 1). In dogs, cranial translation of the tibia relative to the femur ranging from 2.4–16.2 mm has been reported when passively flexing the stifle 120° (21), and in human knees the maximum caudal translation of the tibia relative to the femur ranged from 20 mm – 34 mm, when flexing the joint 100° (27). With the femur in all orientations the medial condyle translated more than the lateral condyle in both flexion and extension which supports the inward/outward rotation of the tibia during flexion/extension of the stifle. With the femur orientated horizontally and upside-down cutting the CCL permitted an increase in cranial/caudal translation tibia relative to the femur under the weight of the tibia and sensors.

The stifle functions mainly as a hinge joint. However, differences in the radius and the length of the articular surface of the femur and tibia result in a complex movement that includes sliding, rolling and rotation of the articular surfaces of the femur and tibia as the stifle flexes and extends (27). The inward/outward rotation of the tibia relative to the femur has been attributed to the difference in length of the medial and lateral femoral condyle with the medial condyle being longer than the lateral condyle. As seen in this study, translation of the medial condyle of the tibia exceeds that of the lateral tibial condyle resulting in internal rotation of the tibia relative to the femur. It is likely that it is the difference in length of the femoral condyles which results in the lateral collateral ligament becoming lax while the cranial component of the medial collateral ligament remains taut (36). The taut medial collateral ligament has a greater influence on the medial aspect of the tibia causing increased translation of the medial aspect of the tibia as the stifle is flexed. The resultant internal rotation of the tibia relative to the femur causes the cruciate ligaments to wrap around one another as well as spiral in on themselves limiting internal rotation during flexion of the stifle.

Limitations of this study were the use of cadaveric limbs stripped of soft tissue, the number and weight of the sensors attached to the tibia, the application of loads to the tibia to cause flexion/extension of the stifle, and resting the tibia on a dowel when taking the measurements. Results from a cadaveric specimen stripped of soft tissue cannot be extrapolated directly to the live patient. However, studies such as these are important in guiding the testing of clinical cases of ruptured CCL. Important insights into the positioning of the leg during testing of clinical cases was gained in this study. It is possible that the weight of the sensors placed in close proximity to the joint may have affected the movement of the tibia relative to the femur. Our initial intention was to measure the rotations and translations of the medial and lateral tibial condyle independently, however, as it is unlikely that the tibia deformed during testing it was decided to use the average of all three sensors to improve the accuracy of the data. Ideally, data should have been obtained from one sensor placed away from the joint. Use of a pulley to flex the stifle and placing the tibia on a dowel during the collection of data likely improved the consistency of testing but may also have introduced a consistent error. We assumed that these forces would be small and would have a negligible effect on the results.

In summary, we have shown that passively flexing/extending the intact and CCL deficient stifle orientated to approximate a standing position, results in movement along a similar paths. Flexion/extension and internal/external rotation are coupled which allows the angle of internal/external rotation of the tibia relative to the femur, in large dogs, to be determined from the flexion/extension angles reported in this study.

## Data availability statement

The original contributions presented in the study are included in the article/supplementary material, further inquiries can be directed to the corresponding author.

## Ethics statement

The animal study was reviewed and approved by the institutional animal care and use committee (#KSVM VTH/31_2019). Written informed consent was obtained from the owners for the participation of their animals in this study.

## Author contributions

JM contributed to conception of study, study design, acquisition of data, and data analysis and interpretation. RB-A, NY, and CY contributed to data analysis and interpretation. All authors drafted, revised and approved the submitted manuscript.

## Conflict of interest

The authors declare that the research was conducted in the absence of any commercial or financial relationships that could be construed as a potential conflict of interest.

## Publisher’s note

All claims expressed in this article are solely those of the authors and do not necessarily represent those of their affiliated organizations, or those of the publisher, the editors and the reviewers. Any product that may be evaluated in this article, or claim that may be made by its manufacturer, is not guaranteed or endorsed by the publisher.
